# Effectiveness of Vortioxetine in Relieving Chronic Pain in Patients with Associated Depression in a Spanish Population

**DOI:** 10.3390/jcm14134487

**Published:** 2025-06-25

**Authors:** Jordi Folch Ibáñez, Maribel Vargas Domingo, Joan Coma Alemany, Roger Callao Sánchez, Jordi Guitart Vela

**Affiliations:** 1 Unit of Pain Pathology, Department of Anesthesiology, Resuscitation and Pain Management, Hospital Plató, 08006 Barcelona, Spain; 2 Unit of Pain Pathology, Department of Anesthesiology, Resuscitation and Pain Management, Parc Sanitari Sant Joan de Déu, Sant Boi de Llobregat, 08830 Barcelona, Spain; 3 Department of Anesthesiology, Resuscitation and Pain Management, Consorci Sanitari Integral, Hospitalet de Llobregat, 08906 Barcelona, Spain; 4 Centre d’atenció i Seguiment de les Drogodependiencis de l’Alt Penedès, Hospital Sagrat Cor, Martorell, 08760 Barcelona, Spain

**Keywords:** chronic pain, depression, observational study, pain, vortioxetine

## Abstract

**Background/Objectives**: The joint presence of chronic pain (CP) and depression is frequent, exacerbating symptoms of both conditions. Although tricyclic antidepressants and serotonin noradrenaline reuptake inhibitors are effective treatments, they are frequently not well tolerated, and selective serotonin reuptake inhibitors are not useful for controlling CP. This study investigated vortioxetine’s effectiveness in relieving CP in patients with any degree of depression. **Methods**: Patient data with any degree of depression and with CP (Visual Analog Scale [VAS] score ≥ 4) were collected and analyzed. Included patients (n = 142) were initially treated with vortioxetine 10 mg/day for 3 months. Improvement of patients’ pain and condition was measured with the VAS, Patient Global Impression (PGI), and Clinical Global Impression (CGI) scales at 1 and 3 months. Brief Pain Inventory (BPI) was measured at baseline and 3 months. Additionally, at baseline and after 3 months of treatment, the Satisfaction with Medicines Questionnaire (SATMED-Q) and 9-item Patient Health Questionnaire (PHQ-9) were evaluated. Adverse Events (AEs) were recorded. **Results**: Patients showed significant improvement (*p* < 0.001) in VAS from baseline to 1 and 3 months (mean [SD]: 7.19 [0.62], 6.23 [0.80], and 5.41 [1.15], respectively). BPI and PHQ-9 scores also showed a significant decrease from baseline (mean [SD] of 6.05 [0.75] and 11.73 [4.89], respectively) to 3 months (5.11 [1.04] and 6.95 [2.52], respectively). Clinical improvement with the CGI and PGI scales were reported. According to the SATMED-Q, patients were satisfied with the treatment. Only a few mild EAs were recorded. **Conclusions**: Vortioxetine can improve both the severity and intensity of CP in patients with any degree of depression.

## 1. Introduction

Chronic pain (CP) is defined as persistent or recurrent pain lasting longer than three months and is a significant source of suffering for patients, thus requiring specific management [1]. CP is a state of stress that affects 20% of the global population [2]. Furthermore, CP is a key contributor in determining depression [3], which is the most common psychological complication in these patients [4,5]. The joint presence of CP and depression exacerbates the symptoms of both conditions, thereby complicating the management of these patients. It has been reported that patients with CP suffering from depression present with a reduced pain threshold, increased pain perception, greater functional limitations, and worse analgesic response [4].

Observations support the idea of overlapping mechanisms that trigger pain- and depression-induced neuroplasticity [3]. These observations include that pain and mood disorders modulate neuronal pathways involved in the same brain regions [6] and are associated with the imbalance of brain-derived neurotrophic factor (BDNF), neurotransmitter norepinephrine (NE, also known as noradrenaline, NA, and serotonin (5-hydroxytryptamine or 5-HT)) levels [7], and, in the central nervous system, alterations in serotonergic neurotransmission [8]. In addition, evidence indicates that serotonergic pathways descending from the rostral ventromedial medulla to the spinal cord are involved in the inhibitory control of pain [4,9]. However, 5-HT’s role in spinal cord pain modulation can be either inhibitory or facilitatory, depending on the predominant receptor subtypes activated. Pharmacological studies have shown that 5-HT-induced analgesia involves 5-HT7 receptors, whereas hyperalgesia involves 5-HT3 receptors [4,9]. While 5–HT–mediated inhibition of nociceptive transmission tends to dominate in acute pain, 5-HT-induced hyperalgesia contributes to the development of chronic pain after tissue or nerve injury [4,10].

Thus, the treatment of these patients with antidepressants with a mechanism of action based on the regulation of neurotransmitters NE and 5-HT could be useful [11,12] for both, alleviating psychological distress in patients with CP and the modulation of pain and inflammation [13,14]. However, the mechanism of action by which antidepressants could reduce inflammation is, to date, still a matter of debate, and further research is needed. On the other hand, tricyclic antidepressants (TCAs) and serotonin noradrenaline reuptake inhibitors (SNRIs) have been shown to be effective in patients with depression and other psychosomatic disorders [12] and the management of concomitant CP [7,8,15]. However, some patients have a poor tolerance to these antidepressants. On the other hand, although selective serotonin reuptake inhibitors (SSRIs) are effective in patients with depression [15,16], they are not useful in the management of neuropathic pain [17]. Therefore, treatment alternatives for patients with CP and psychological complications remain insufficient.

The antidepressant vortioxetine (marketed as Brintellix^®^ by H. Lundbeck A/S, Denmark) presents a multimodal mechanism of action. It is indicated in adults with a good safety and tolerability profile [18]. According to the technical data sheet [19,20], vortioxetine’s maximum plasma concentration (Cmax) is reached between 7 and 11 h, with mean Cmax values of 9 to 33 ng/mL observed. Absolute bioavailability is 75%. The mean volume of distribution (Vss) is 2600 L, indicating a wide extravascular distribution. Vortioxetine is highly bound to plasma proteins (98 to 99%), independently of its plasma concentrations. It is extensively metabolized in the liver, mainly by oxidation catalyzed by CYP2D6 and, to a lesser extent, by CYP3A4/5 and CYP2C9, and subsequent conjugation to glucuronic acid. The elimination half-life and oral clearance are 66 h and 33 L/h, respectively. Approximately 2/3 of the inactive metabolites of vortioxetine are eliminated in the urine, and approximately 1/3 in the feces (only negligible amounts). Steady-state plasma concentrations are reached in approximately 2 weeks.

Vortioxetine has already demonstrated clinical efficacy in major depressive disorder (MDD) [21,22,23,24], on physical [25], affective [26], and anxiety symptoms [27,28,29]. In addition, Vortioxetine has also been shown to improve cognitive dysfunction associated with unipolar depression [21,22,24,26,30] and dementia [22]. Recently, many preclinical and clinical studies have proved that vortioxetine may be useful in the treatment of CP [8,13,31,32,33]. However, further evidence is required to confirm this.

Vortioxetine can bind to the serotonin transporter (SERT) with high affinity (Ki 1.6 nM), inducing the inhibition of its activity and the increase in 5-HT levels [34]. Furthermore, vortioxetine is considered an enhancer of serotonergic transmission and a potent inhibitor of 5-HT3 receptors, as it regulates multiple 5-HT receptor subtypes. It exerts partial agonist action at 5-HT1B receptors (Ki = 33 nM), agonist action at 5-HT1A receptors (with Ki = 15 nM), and antagonist action at 5-HT1D (with Ki = 54 nM), 5HT3 (with Ki = 3.7 nM), and 5HT7 (with Ki = 19 nM) receptors [34,35]. However, although the clinical relevance of each individual receptor interaction is not yet fully understood, taking into account the possible involvement of 5-HT3 receptors in 5HT-induced hyperalgesia, vortioxetine could be an interesting alternative with which to treat CP because of its capacity to inhibit 5-HT3 receptors [4,33].

Recently, an observational study was conducted in patients diagnosed with MDD and associated CP conditions (Visual Analogic Scale [VAS] score ≥ 4) [36]. The results showed that treatment with 10 mg/day of vortioxetine for 3 months not only significantly reduced depression but also the CP suffered by these patients. Therefore, additional data from patients with any degree of depression and treated with vortioxetine were collected from the researchers’ databases and were added to the patient data from the previous study to evaluate the efficacy of vortioxetine in relieving CP (VAS score ≥ 4) in patients with any degree of depression. Thus, we aimed to provide further evidence regarding the analgesic capacity of vortioxetine in the treatment of CP.

## 2. Materials and Methods

### 2.1. Study Design and Patients

Data obtained from patients with MDD treated with vortioxetine who participated in the previous observational study conducted in Barcelona, Spain, from 5 November 2020, to 23 February 2023 [36] and from the researchers’ database on patients with any degree of depression were collected and analyzed. All patients provided written informed consent before participating in the study. The design, patients (including inclusion and exclusion criteria), and methods have been described previously [36]. Thus, in this study, patients with any degree of depression, and not only with MDD (as indicated in the inclusion criteria of Folch et al. [36]), evaluated using the 9-item Patient Health Questionnaire (PHQ-9), were considered. All patients’ data collected for this study followed the same procedures (follow-up, treatment regimen, evaluation, etc.) and were obtained from the same centers.

Data were collected from patients attending outpatient pain units and showed a partial response to their current medication and/or experienced significant adverse effects. All patients showed a CP condition (VAS score ≥ 4) and associated depression.

The vortioxetine baseline treatment was 10mg/day. When possible, fifteen days after initiating the treatment with vortioxetine, patients were contacted by phone. Depending on their individual clinical evolution and according to medical judgment, the vortioxetine dose could be escalated to the highest recommended dose of 20 mg/day or decreased to the minimal recommended dose of 5 mg/day following the approved MDD dosing guidelines (5–20 mg/day) [19]. In case of discontinuation of treatment with vortioxetine, no stepwise withdrawal was planned. As per common clinical practice, all patients continued to receive their usual pain medication during the study. Assessments and follow-ups were performed, as previously described [36], during 3 visits: at baseline (visit 1), at 1 month (±2 weeks) of treatment (visit 2), and at 3 months (±2 weeks) of vortioxetine treatment (visit 3).

### 2.2. Study Assessments

This data collection analysis aimed to assess pain improvement using the VAS scale [37] and Brief Pain Inventory (BPI) [38]. Improvements in depressive symptoms (PHQ-9) [39] and the Chronic Pain Coping Inventory (in Spanish, “Cuestionario de Afrontamiento del Dolor” [CAD]) [40] were also evaluated. Overall disease severity was assessed using the Patient Global Impression Improvement (PGI-I) scale [41] and the Clinical Global Impression (CGI) scale [42], and patients’ satisfaction was evaluated using the Satisfaction with Medicines Questionnaire (SATMED-Q) [43]. All the scales and questionnaires were described and implemented as previously described [36].

Adverse Events (AEs) associated with vortioxetine were described during treatment, including the start and end dates, outcome, causality relationship, and severity criteria.

The PHQ-9 questionnaire, BPI scale, and CAD and SATMED-Q questionnaires were administered at visits 1 and 3. The VAS, PGI, and CGI scales were administered at visits 1, 2, and 3.

### 2.3. Statistical Analysis

All eligible participants who had received at least one dose of vortioxetine and presented at least one post-baseline data point assessment were considered evaluable. Patients who discontinued the treatment were excluded. The safety population contained all patients who received at least one vortioxetine dose.

Statistical analyses were similar to those used in a previous study [36]. Continuous variables were described by the number of valid cases, mean, and standard deviation (SD). Also, the median, minimum, maximum, and 25th and 75th percentiles (P25–P75) were described. Descriptive statistical analyses were used to assess all variables. Categorical variables were described by the absolute and relative frequencies of each category over the total number of valid values (n). Variables used to evaluate the reduction in pain, depressive symptoms, and overall disease severity were analyzed using Student’s *t*-test for paired data, justified by the sample size and, thus, assuming normality in the data. Patient satisfaction and AEs were analyzed using the chi-squared test. For all comparisons, a bilateral statistical significance level of 0.05 was used.

Since this study is a one-arm study assessing changes from baseline, patients served as their own control, which reduces the risk of confounding.

Statistical analysis was performed following the principles specified in the ICH E9 guidelines and the standards of good clinical practice. Statistical analysis was performed using SAS (Statistical Analysis System), version 9.4, on a Windows platform.

## 3. Results

### 3.1. Population

The data came from the 64 patients who participated in the previous observational study [36] (with MDD), and from 78 patients registered in the researchers’ medical records (with any degree of depression). Therefore, data from a total of 142 patients were included in this study. All of them (100%) signed the informed consent form and completed the study.

The mean age of the participants was 65.5 years old. The majority of patients were women (78.2%). Only five participants (3.5%) reported to be on medical leave. All patients suffered from some degree of depression, and most of them (93.0%) reported at least one comorbidity, with hypertension being the most common. All of them had previously received treatment for pain; the most common were paracetamol (68.3%), NSAIDs (63.4%), and pregabalin (40.1%). Furthermore, 97.2% of them reported previous treatment with antidepressants, with the most common ones being escitalopram (27.5%) and duloxetine (21.8%). Table 1 shows the baseline demographics and characteristics.

Most patients (93.0%) experienced severe pain. The mean intensity of pain (SD) was 7.33 (0.79). Depending on the time of evolution, 33.8% of patients suffered from pain lasting >3 months and <12 months, and 31.7% with pain lasting >2 years and <5 years. The pain location was mainly in the lower extremities (69.0%), followed by lower back in 64.8% of the participants. Patients also reported mixed pain (94.4%) as the most common one. The most frequent origins of the pain were lumbosciatica (43.0%), osteoarthritis (40.8%), and degenerative spinal problems (32.4%). Patients already developed coping strategies to manage their pain, most commonly through distraction, readiness to find information, and self-affirmation (Table S1). According to the reported baseline clinical assessments (Table 2), patients suffered moderate (73.2%) to severe (26.8%) pain that interfered with their daily life activities.

The starting dose for all participants was 10 mg/day. Six patients received an increased dose (20 mg/day) in visits 2 and 3 (Table 3). At 3 months, the mean (SD) vortioxetine dose was 10.42 (2.02) mg/day.

### 3.2. Effect of Vortioxetine on Pain

**Figure 1:**
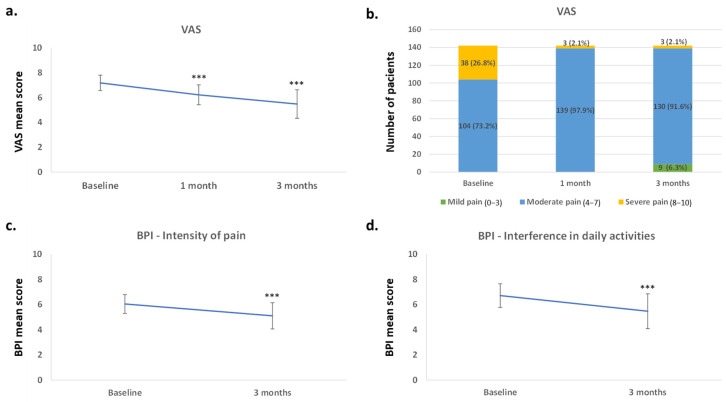
Pain variations, from baseline to 1 and 3 months, measured by VAS (**a**,**b**) and BPI (**c**,**d**) scores: (**a**) mean VAS score ± SD and (**b**) percentage and number of patients according to intensity of pain, at each time point; (**c**) intensity of pain and (**d**) interference of pain in daily activities, according to BPI scale ± SD at each time point. *** *p* < 0.0001 with respect to the baseline. BPI, Brief Pain Inventory; SD, standard deviation; VAS, Visual Analogic Scale.

The VAS score decreased over the 3-month treatment period, with significant reductions from baseline observed at both visits 2 and 3 (mean [SD]: 7.19 [0.62], 6.23 [0.80], and 5.41 [1.15], respectively [*p* < 0.0001]) (Figure 1a). At baseline (visit 1), 73.2% of the patients experienced moderate pain and 26.8% severe pain; most of them presented moderate pain (97.9%) at visit 2; and, at visit 3, they reported mild to moderate pain (6.3% and 91.5%, respectively) (Figure 1b), indicating that the intensity of pain was decreasing. Only three patients (2.1%) reported severe pain at 3 months.

At 3 months, severity of pain, evaluated by the BPI scale (Figure 1c), showed a significant decrease in the patients (mean [SD] of 6.05 (0.75) at baseline and 5.11 (1.04) at 3 months [*p* < 0.0001]). In addition, the interference of pain with daily activities decreased significantly (Figure 1d), with a mean [SD] score at baseline of (6.72 [0.94]) that was reduced at 3 months (5.48 [1.38]) (*p* < 0.0001).

### 3.3. Effect of Vortioxetine in the Overall Disease Severity and on Depressive Symptoms

**Figure 2:**
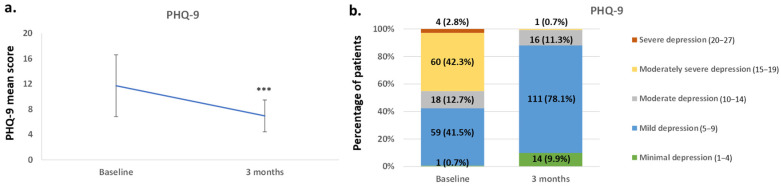
Changes in the PHQ-9 score at 3 months from baseline, in the patients after treatment with vortioxetine. Measures were expressed as (**a**) mean ± SD and (**b**) according to the severity of depression at each time point, with the percentage and number of patients. *** *p* < 0.0001 with respect to the baseline. SD: standard deviation.

The PHQ-9 score showed a statistically significant clinical recovery in the severity of depressive symptoms at three months (*p* < 0.0001) (Figure 2). The PHQ-9 score varied from 11.73 (4.89) at baseline to 6,95 (2.52) at 3 months (Figure 2a). After the 3 months of treatment, 78.2% of patients reported mild depression (n = 111), 11.6% moderate depression (n = 16), and 9.9% minimal depression (n = 14) (Figure 2b).

**Figure 3:**
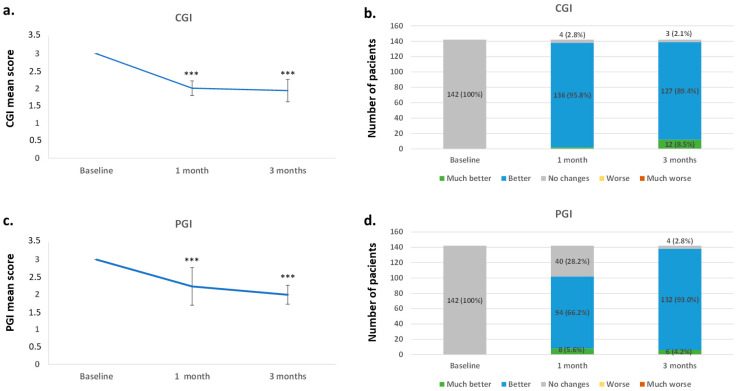
Improvement of the patients evaluated with CGI and PGI scores from baseline to 1 and 3 months: (**a**) mean CGI score ± SD; (**b**) improvement of the patients (number and percentage) according to the CGI score at each time point; (**c**) mean PGI score ± SD; (**d**) percentage and number of patients reporting improvement at each time point. *** *p* < 0.0001 with respect to the baseline. CGI, Clinical Global Impression; PGI: Patient Global Impression; SD, standard deviation.

The CGI score pointed to a significant decrease (*p* < 0.0001 in all time points), meaning an improvement in patients’ condition from baseline to 1 and 3 months (Figure 3a,b). The CGI score mean (SD) was 3.00 (0.00) at baseline, 2.01 (0.21) at 1 month, and 1.94 (0.32) at 3 months. Clinicians also perceived 89.4% of the patients as better and 8.5% of them as much better at 3 months. The PGI mean (SD) score showed, at both 1 and 3 months, a significant (*p* < 0.0001) improvement (2.23 [0.54] 1.99 [0.27], respectively) from baseline (3.00 [0.00]). In addition, 93.0% of the patients felt better after the 3 months of treatment, while 4.2% felt much better (Figure 3c,d).

### 3.4. Safety, Tolerability, and Acceptance

AEs were reported in 28.9% and 29.6% of the patients at 1 and 3 months, respectively, and all of them were mild. Four types of AEs were reported and ranked as follows: sleepiness (37 [26.1%] and 36 [25.4%] patients, respectively), dry mouth (4 [2.8%] and 5 [3.5%] patients, respectively), nausea (1 [0.7%] patient at week 2), and headache (1 [0.7%] patient at 3 months). No other AEs were reported.

In addition, patients were satisfied with vortioxetine treatment, as informed by the SATMED-Q questionnaire, with no safety concerns reported (Table S2).

## 4. Discussion

CP is a stress state affecting 20% of the global population [2], whose most common psychological complication is depression [4]. The joint presence of CP and depression is a complicated state in which the symptoms of both conditions are exacerbated. Thus, the management of these patients is very complicated, with a reduced pain threshold, increased pain perception, worse analgesic response, and more significant functional limitations [4].

In these cases, antidepressants can be useful because they can regulate 5-HT and NE, neurotransmitters involved in the pathophysiology underlying CP [11]. Although TCAs and SNRIs are effective first-line treatments [7,8,15], they produce AEs, and SSRIs present little clinical evidence in CP [17]. Vortioxetine is an interesting alternative for treating patients with CP since, recently, its clinical effectiveness in relieving pain and symptoms of depression, with a very good safety profile, has been shown [13,31,36].

The population from which data were collected and analyzed comprised outpatients attending pain units with a condition of CP and diagnosed with associated depression, also receiving concomitant treatment for pain, but never treated before with vortioxetine. Most of them received treatment for depression too. Vortioxetine was prescribed following the approved MDD dosing guidelines (5–20 mg/day) [19]. The results of this analysis not only confirm, as expected, that vortioxetine reduces depressive symptoms with a good safety profile but also show it to be significantly effective in pain management in patients with CP; thus, while most patients at baseline reported moderate to severe pain, at 3 months, this pain became moderate to mild for most of the patients, accompanied by a decrease in its severity and its interference with daily activities.

In this data collection, additional data from patients with any degree of depression and treated with vortioxetine were collected from the researchers’ databases and were added to those obtained from patients with MDD in the context of our previously conducted observational study (Folch et al.) [36]. The decrease in the severity of pain was indicated by a decrease of 2 points in the VAS score at 3 months, compared to baseline, as previously reported [13,31]. Furthermore, this decrease was already observed by Folch et al. [36]. In addition, according to the BPI scale, a decrease of 1 and 1.24 points was observed at 3 months for pain intensity and pain interfering with daily life activities. Both Folch et al. [36] and the current data collection demonstrate that patients have different types of pain at different locations originating from different causes, which reinforces the effectiveness of vortioxetine in decreasing pain in any type of CP in a reproducible manner. However, we noticed that the decrease in the BPI scale score shown in this data collection was lower than that observed by Folch et al. [36]., which was 1.5 and 2 points for the intensity of pain and interference with daily activities, respectively. This could be explained by the higher scores observed at baseline in our previous study, 6.20 vs. 6.05 for pain intensity and 7.02 vs. 6.72 for interference with daily activities. Thus, the drop in score would also be smaller, especially for the results of interference with daily activities. In addition, in this study, the treatment with vortioxetine followed usual clinical practice; thus, no samples were taken from patients to analyze the pharmacokinetic profile when administering vortioxetine. Therefore, we cannot determine whether the lower decrease in the BPI scale in these patients compared with other studies may have been due to pharmacokinetic factors. However, we believe it is unlikely, since vortioxetine was prescribed following the approved MDD dosing guidelines (5–20 mg/day) [19].

Despite this, it is important to note the clinical efficacy of vortioxetine reported using both the CGI and PGI scales. Both clinicians and patients perceived an improvement in the participants’ overall disease severity and its impact on global functioning. This improvement on both scales was similar to that observed by Folch et al. [36]. In addition, the positive results obtained with the CGI score after treatment with vortioxetine have already been reported in previous studies [31]. Participants also reported in the SATMED-Q that they felt better and satisfied with vortioxetine treatment.

As expected, most patients improved in their depressive symptoms. The PHQ-9 questionnaire’s mean score decreased by 4 points at 3 months compared to baseline, and most patients manifested mild depression (78.2%). The effectiveness of vortioxetine in improving depressive symptoms has been previously reported [21,22,23,24,31]. However, this reduction was lower than that observed by Folch et al. [36], which reported 9 points of improvement. However, in the previous study, all patients started with PHQ-9 ≥ 15 (mean [SD]: 16.63 [1.47]), while in this data collection, depression was considered an additional comorbidity, with a mean PHQ-9 score (SD) of 11.73 (4.89). Thus, the decrease in punctuation was expected to be lower.

The CP and depression binomial should also be considered in this context. All patients suffered from both CP and depression before starting vortioxetine treatment. All patients had previously received treatment for pain, and 97.2% had received treatment for depression. The present data collection proved that when patients use their pain medication at the same time as vortioxetine instead of their previous antidepressant, pain management improves. These results show, like previously [36], that vortioxetine has been crucial for the decrease in CP symptoms, accompanied by general improvement in the patients.

One limitation of this study was its short duration. In addition, it was a single-arm, nonrandomized data collection, and no assessment of patient functioning was used, which is important for both depression and CP. In addition, changes in concomitant treatment in the case of a suboptimal response that could explain the lower therapeutic effect of treatment with vortioxetine were not collected. However, the data collection was performed in the context of real clinical practice. It demonstrates that treatment with vortioxetine can improve the severity and intensity of CP from any cause in patients with any degree of depression.

## Figures and Tables

**Table 1:** Baseline patient demographics and clinical characteristics.

Characteristic	n (%)
Male/Female	31 (21.8%)/111 (78.2%)
Employment status	
Total	142 (100.0%)
Active	58 (40.8%)
Unemployed	-
Medical leave	5 (3.5%)
Permanent incapacity for work	-
Retired	79 (55.6%)
Comorbidities	
With any comorbidity	132 (93.0%)
AHT	94 (66.2%)
Osteoporosis	54 (38.0%)
Hypothyroidism	21 (14.8%)
Cardiovascular disease	19 (13.4%)
Diabetes mellitus	16 (11.3%)
Prostate pathology	6 (4.2%)
Respiratory disease	6 (4.2%)
Hyperthyroidism	1 (0.7%)
Other	1 (0.7%)
Intensity of the pain	
No pain	-
Mild	-
Moderate	6 (4.2%)
Severe	132 (93.0%)
Unbearable	3 (2.1%)
Missing	1 (0.7%)
Location of the pain	
Lower extremities	98 (69.0%)
Lumbar	92 (64.8%)
Shoulders	19 (13.14%)
Head neck	7 (4.9%)
Dorsal	4 (2.8%)
Upper extremities	4 (2.8%)
Type of pain	
Somatic	2 (1.4%)
Visceral	-
Neuropathic	6 (4.2%)
Mixed	134 (94.4%)
Psychogenic	2 (1.4%)
Others	3 (2.1%)
Main cause of chronic pain	
Lumbosciatica	61 (43.0%)
Arthrosis	58 (40.8%)
Degenerative problems of the spine	46 (32.4%)
Fibromyalgia	13 (9.2%)
Peripheral neuropathy	7 (4.9%)
Herpes zoster	3 (2.1%)
Complex regional syndrome	1 (0.7%)
Tumor	-
Visceral	-
Other	1 (0.7%)
Time of evolution	
>3 months and <12 months	48 (33.8%)
>12 months and <24 months	30 (21.1%)
>2 years and <5 years	45 (31.7%)
>5 years	19 (13.4%)
Previous treatments for depression	
Escitalopram	39 (27.5%)
Duloxetine	31 (21.8%)
Citalopram	19 (13.4%)
Venlafaxine	15 (10.6%)
Amitriptyline	11 (7.7%)
Sertraline	7 (4.9%)
Bupropion	4 (2.8%)
Paroxetine	1 (0.7%)
Fluoxetine	1 (0.7%)

AHT, arterial hypertension.

**Table 2:** Patients’ baseline clinical assessments using the PHQ-9 and VAS scales.

Variable	n (%)
PHQ-9	
Minimum depression (1–4)	1 (0.7%)
Mild depression (5–9)	59 (41.5%)
Moderate depression (10–14)	18 (12.7%)
Moderately severe depression (15–19)	60 (42.3%)
Severe depression (20–27)	4 (2.8%)
VAS	
Mild pain (0–3)	-
Moderate pain (4–7)	104 (73.2%)
Severe pain (8–10)	38 (26.8%)

PHQ-9, 9-item Patient Health Questionnaire; VAS, Visual Analogic Scale.

**Table 3:** Dose prescription of vortioxetine.

	Vortioxetine 5 mg/day n (%)	Vortioxetine 10 mg/day n (%)	Vortioxetine 15 mg/day n (%)	Vortioxetine 20 mg/day n (%)
Baseline (n = 142)	-	142 (100)	-	-
1 month (n = 142)	-	136 (95.77)	-	6 (4.23)
3 months (n = 142)	-	136 (95.77)	-	6 (4.23)

mg, milligram.

## Data Availability

The dataset is available on request from the authors.

## Supplementary Materials

The following supporting information can be downloaded at https://www.mdpi.com/article/10.3390/jcm14134487/s1: Table S1: Chronic Pain Coping Inventory (in Spanish, “Cuestionario de Afrontamiento del Dolor” [CAD]): results from the 142 participants; Table S2: Satisfaction with Medicines Questionnaire (SATMED-Q): results from the 142 participants.
