# Supplementary material for: Effectiveness of Vortioxetine in Relieving Chronic Pain in Patients with Associated Depression in a Spanish Population
Source: J Clin Med. 2025 Jun 25;14(13):4487. doi: 10.3390/jcm14134487 (PMC12250573; doi:10.3390/jcm14134487)
Supplement: Supplementary file 1 [file jcm-14-04487-s001.zip › jcm-3684650-supplementary.pdf]

Table S1: Chronic Pain Coping Inventory (in Spanish, "Cuestionario de Afrontamiento del Dolor" [CAD]): results from the 142 participants.

**Dimension**	**Mean (SD)**
Religion	12.40 (5.24)
Catharsis	18.16 (2.97)
Distraction	18.78 (2.53)
Mental self-control	16.96 (2.56)
Self-assertion	18.77 (2.23)
Readiness to find information	20.68 (1.82)

Table S2: Satisfaction with Medicines Questionnaire (SATMED-Q): results from the 142 participants.

Items	Visit 1	Visit 3	*P*
Undesirable effects			
1. The side effects of medication interfere with my physical activity.			
No, nothing	128 (90.1%)	131 (92.9%)	0.5474
Some	6 (4.2%)	3 (2.1%)	
Not very much, not a little	5 (3.5%)	6 (4.3%)	
Quite a lot	3 (2.1%)	1 (0.7%)	
Yes, a lot	-	-	
N missing	0	1	
2. The side effects of the medication interfere with my leisure and free time activities.			
No, nothing	129 (90.8%)	132 (93.6%)	0.2107
Some	6 (4.2%)	2 (1.4%)	
Not very much, not a little	4 (2.8%)	7 (5.0%)	
Quite a lot	2 (1.4%)	-	
Yes, a lot	1 (0.7%)	-	
N missing	0	1	
3. The side effects of the medication interfere with my daily tasks.			
No, nothing	128 (90.1%)	131 (92.9%)	0.6731
Some	6 (4.2%)	3 (2.1%)	
Not very much, not a little	4 (2.8%)	5 (3.5%)	
Quite a lot	3 (2.1%)	2 (1.4%)	
Yes, a lot	1 (0.7%)	-	
N missing	0	1	
Effectiveness of medication			

4. The medication I am taking relieves my symptoms.<br><br>No, nothing<br>Some<br>Not very much, not a little<br>Quite a lot<br>Yes, a lot<br>N missing	 <br><br>4 (2.8%)<br>48 (33.8%)<br>20 (14.1%)<br>69 (48.6%)<br>1 (0.7%)<br>0	 <br><br>-<br>41 (29.1%)<br>13 (9.2%)<br>86 (61.0%)<br>1 (0.7%)<br>1	0.0954
5. I am satisfied with the time it takes for the medicine to start working.<br><br>No, nothing<br>Some<br>Not very much, not a little<br>Quite a lot<br>Yes, a lot<br>N missing	 <br><br><br>4 (2.8%)<br>30 (21.1%)<br>39 (27.5%)<br>68 (47.9%)<br>1 (0.7%)<br>0	 <br><br><br>-<br>21 (14.9%)<br>49 (34.8%)<br>69 (48.9%)<br>2 (1.4%)<br>1	0.1327
6. I feel better now than I did before I started the treatment.<br><br>No, nothing<br>Some<br>Not very much, not a little<br>Quite a lot<br>Yes, a lot<br>N missing	 <br><br>-<br>37 (26.1%)<br>28 (19.7%)<br>73 (51.4%)<br>4 (2.8%)<br>0	 <br><br>-<br>28 (19.9%)<br>16 (11.3%)<br>90 (63.8%)<br>7 (5.0%)<br>1	0.0686
Convenience of the medication			
7. I find it comfortable to take my medication<br><br>No, nothing<br>Some<br>Not very much, not a little<br>Quite a lot<br>Yes, a lot<br>N missing	 <br><br>1 (0.7%)<br>10 (7.0%)<br>18 (12.7%)<br>105 (73.9%)<br>8 (5.6%)<br>0	 <br><br>-<br>5 (3.5%)<br>24 (17.0%)<br>101 (71.6%)<br>11 (7.8%)<br>1	0.3964
8. I find it easy to use/take the medicine in its current form (taste, size, etc.).<br><br>No, nothing<br>Some<br>Not very much, not a little<br>Quite a lot<br>Yes, a lot<br>N missing	 <br><br><br>1 (0.7%)<br>4 (2.8%)<br>53 (37.3%)<br>77 (54.2%)<br>7 (4.9%)<br>0	 <br><br><br>-<br>1 (0.7%)<br>47 (33.3%)<br>82 (58.2%)<br>11 (7.8%)<br>1	0.3793
9. I find the schedule of the shots convenient			0.4860

No, nothing	1 (0.7%)	-	
Some	8 (5.6%)	4 (2.8%)	
Not very much, not a little	23 (16.2%)	26 (18.4%)	
Quite a lot	103 (72.5%)	100 (70.9%)	
Yes, a lot	7 (4.9%)	11 (7.8%)	
N missing	0	1	
Impact of medication			
10. Thanks to the medication I am taking, I am better able to carry out my leisure and free time activities.			0.2498
No, nothing	-	-	
Some	35 (24.6%)	30 (21.3%)	
Not very much, not a little	20 (14.1%)	11 (7.8%)	
Quite a lot	86 (60.6%)	98 (69.5%)	
Yes, a lot	1 (0.7%)	2 (1.4%)	
N missing	0	1	
11. Thanks to my medication I am better able to perform my personal grooming tasks.			0.0080
No, nothing	2 (1.4%)	1 (0.7%)	
Some	31 (21.8%)	28 (19.9%)	
Not very much, not a little	48 (33.8%)	24 (17.0%)	
Quite a lot	60 (42.3%)	85 (60.3%)	
Yes, a lot	1 (0.7%	3 (2.1%)	
N missing	0	1	
12. Thanks to my medication I can perform my daily tasks better.			0.0023
No, nothing	-	-	
Some	34 (23.9%)	26 (18.4%)	
Not very much, not a little	27 (19.0%)	10 (7.1%)	
Quite a lot	80 (56.3%)	99 (70.2%)	
Yes, a lot	1 (0.7%)	6 (4.3%)	
N missing	0	1	
Medical follow-up			
13. My doctor has informed me in detail about my illness.			0.2527
No, nothing	-	-	
Some	-	-	
Not very much, not a little	6 (4.3%)	2 (1.4%)	
Quite a lot	38 (27.0%)	33 (23.4%)	
Yes, a lot	97 (68.8%)	106 (75.2%)	
N missing	1	1	
14. My doctor has informed me about how to treat my illness correctly.			0.1494

No, nothing<br>Some<br>Not very much, not a little<br>Quite a lot<br>Yes, a lot<br>N missing	-<br>3 (2.1%)<br>4 (2.8%)<br>35 (24.8%)<br>99 (70.2%)<br>1	-<br>-<br>1 (0.7%)<br>32 (22.7%)<br>108 (76.6%)<br>1	
General view			
15. I intend to continue using this treatment<br><br>No, nothing<br>Some<br>Not very much, not a little<br>Quite a lot<br>Yes, a lot<br>N missing	 <br>-<br>7 (4.9%)<br>10 (7.0%)<br>114 (80.3%)<br>11 (7.7%)<br>0	 <br>-<br>3 (2.1%)<br>4 (2.8%)<br>127 (90.1%)<br>7 (5.0%)<br>1	0.1240
16. I feel comfortable with my treatment<br><br>No, nothing<br>Some<br>Not very much, not a little<br>Quite a lot<br>Yes, a lot<br>N missing	 <br>1 (0.7%)<br>5 (3.5%)<br>28 (19.7%)<br>102 (71.8%)<br>6 (4.2%)<br>0	 <br>-<br>4 (2.8%)<br>17 (12.1%)<br>111 (78.7%)<br>9 (6.4%)<br>1	0.3110
17. Overall, I am satisfied with the treatment<br><br>No, nothing<br>Some<br>Not very much, not a little<br>Quite a lot<br>Yes, a lot<br>N missing	 <br>1 (0.7%)<br>9 (6.3%)<br>12 (8.5%)<br>117 (82.4%)<br>3 (2.1%)<br>0	 <br>-<br>4 (2.8%)<br>5 (3.5%)<br>124 (87.9%)<br>8 (5.7%)<br>1	0.0819
A bilateral statistical significance level of 0.05 was used for all comparisons.			
